# Connective tissue growth factor in tumor pathogenesis

**DOI:** 10.1186/1755-1536-5-S1-S8

**Published:** 2012-06-06

**Authors:** Annica Jacobson, Janet L Cunningham

**Affiliations:** 1 Section of Osteoporosis and Clinical Pharmacogenetics, Department of Medical Sciences, Uppsala University, Uppsala, Sweden; 2 Department of Neuroscience, Psychiatry, Uppsala University, Sweden

## Abstract

Key roles for connective tissue growth factor (CTGF/CCN2) are demonstrated in the wound repair process where it promotes myofibroblast differentiation and angiogenesis. Similar mechanisms are active in tumor-reactive stroma where CTGF is expressed. Other potential roles include prevention of hypoxia-induced apoptosis and promoting epithelial-mesenchymal transistion (EMT). CTGF expression in tumors has been associated to both tumor suppression and progression. For example, CTGF expression in acute lymphoblastic leukemia, breast, pancreas and gastric cancer correlates to worse prognosis whereas the opposite is true for colorectal, lung and ovarian cancer. This discrepancy is not yet understood.

High expression of CTGF is a hallmark of ileal carcinoids, which are well-differentiated endocrine carcinomas with serotonin production originating from the small intestine and proximal colon. These tumors maintain a high grade of differentiation and low proliferation. Despite this, they are malignant and most patients have metastatic disease at diagnosis. These tumors demonstrate several phenotypes potentially related to CTGF function namely: cell migration, absent tumor cell apoptosis, as well as, reactive and well vascularised myofibroblast rich stroma and fibrosis development locally and in distal organs. The presence of CTGF in other endocrine tumors indicates a role in the progression of well-differentiated tumors.

## Background

It is well established that connective tissue growth factor (CTGF/CCN2) is a key component in wound repair. A consensus for the role of CTGF in tumorigenesis, however, has been surprisingly difficult to reach despite studies in many tumor types and cell-lines. Directly opposing effects have been demonstrated in different tumor types and even within the same tumor diagnosis (summarized in Table 1). This review provides a brief summary of our current knowledge and treats possible explanations for discrepancies between studies. Extra focus will be given to ileal carcinoids, which express high levels of CTGF, as their unique phenotype and tumor behavior may provide a model for understanding CTGF function.

### CTGF in development

Tissue-specific developmental programs are often reactivated in solid tumors and understanding CTGF's role in development may be relevant to its role in tumorigenesis. CTGF knockout mice die at birth due to skeletal defects in the ribcage that impair respiration [1]. This phenotype is thought to involve impaired chondrocyte proliferation and disturbed angiogenesis. Interestingly, null embryos also have abnormal pancreatic islet morphology. Specifically, they display higher numbers of glucagon positive cells and fewer insulin positive cells. The CTGF heterozygotes survive past birth and exhibit generally alpha- and beta-cell hypertrophy which indicates that CTGF may be involved in the establishment of normal islet endocrine cell ratio and architecture but their gross appearance is otherwise normal [2,3]. A nine-fold overexpression of CTGF in transgene mice causes abnormalities, including developmental delay and craniofacial defects, and embryonic death but an overt fibrotic phenotype was not seen [3].

### CTGF protein structure and posttranslational modifications

**Figure 1:**
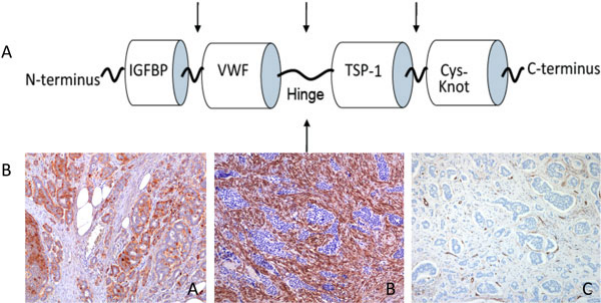
**A: Full length CTGF contains 4 modules: insulin-like growth factor binding protein-like (IGFBP), von Willebrand factor type-C repeat (VWF), thrombospondin type 1 repeat (TSP-1) and C-terminal cystine knot (cys-knot)**. Arrows indicate potential cleavage sites. B: Ileal carcinoid tissue immunostained with antibodies to CTGF (A), α-SMA (B) and CD31/CD34 (C) identifies vascular endothelial cells demonstrating typical tumour cell IR for CTGF and stromal expression of α-SMA detected both in myofibroblasts and in vascular smooth muscle cells.

CTGF is a 349-amino acid polypeptide consisting of four domains (see Figure 1A). Full length CTGF has an estimated molecular weight of 33 kD while cleavage in the hinge region produces N- and C-terminal fragments with an estimated molecular weight of 20-23 kD and these fragments may have specific biological functions [4,5]. Different cellular sources may offer different proteases for CTGF processing, thus forming a subset of fragments, which in turn determines CTGF action. It is speculated that CTGF may even promote its own proteolysis in some tissues, resulting in differential retention of a specific fragment [6]. High levels of N-terminal CTGF are found in patients with fibrotic scleroderma [7] and in diabetic patients with nephropathy [8].

In vitro studies demonstrate that N-terminal CTGF mediates TGF-β induced myofibroblast differentiation by upregulating expression of alpha-smooth muscle actin (α-SMA) and collagen. In the same study, in the presence of C-terminal CTGF, proliferating fibroblast were observed, which neither express α-SMA nor produce collagen [4]. This study also indicates that the presence of other growth factors, such as EGF, IGF-I and II and TGF-β, may influence the degree to which these different fibrotic processes are active [9].

In vivo, full length CTGF is often glycosylated and this increases the molecular weight from 33 kD to 42 kD or more. This glycosylation may be important for function. For example, in a renal fibroblast cell line, overexpression of CTGF results in growth arrest, whereas exogenously added recombinant CTGF increased proliferation [10].

## Methods

We aimed to examine the role of CTGF in tumorigenesis. Medline articles, other reviews and citation lists in relevant articles were searched for studies on CTGF's expression in human tumor specimens, primary human tumor cell cultures, knock-out mice and normal tissue (see supplement for search terms). Articles were published between the years 1997-2010. Studies that only showed results from cell lines were excluded. CTGF expression in tumors tissue was compared to normal tissue. Studies were sorted according to the cancer type and endocrine phenotype and according to reported CTGF correlation to tumor progression.

## Results

### CTGF in tumorigenesis

CTGF's role in tumor development can be divided into 3 categories seen in additional file 1: tumor promotion, suppression or both (complex). There appears to be a relationship between CTGF's role and its basal expression in the original normal cell or non-transformed tissue. Group A tumors, which includes breast and pancreas cancer have a positive correlation between CTGF and tumor development -- higher levels of CTGF are expressed in metastases and CTGF is correlated to later tumor stages. The normal cell counterparts of these tumors are not found to express CTGF. CTGF is normally expressed in endothelial cells, fibroblasts, smooth muscle cells and myofibroblasts, and some epithelial cell types in diverse normal tissues [11]. Group B tumors, which include chondrosarcoma [12] and lung cancer [13], are formed from cell types, which are reported to express CTGF and are often reported to have an inverse relationship between the level of CTGF expression and malignant phenotype (Additional file 1).

The complicated nature of CTGF in tumor progression is evident in the group C tumors. In ovarian cancer, for example, CTGF is normally expressed in apical cytoplasm of normal ovarian epithelial cells. Gene methylation and reduced expression, in relation to normal cells, of CTGF is frequently observed in primary ovarian cancer tissues. High CTGF expression in early stage ovarian tumors appears to have a protective effect procuring prolonged survival but this must be verified in a larger cohort. In vitro studies on a panel of ovarian cancer cell lines support CTGF's putative tumor growth suppressive effect: in cells lacking CTGF, both exogenous restoration of expression and treatment with recombinant CTGF inhibited proliferation while knockdown of CTGF in cells with endogenous expression accelerated proliferation [14]. Surprisingly, CTGF expression is often restored in the most advanced tumor stages of ovarian cancer and is correlated to a more malignant phenotype [14].

Factors other than CTGF differ between the tumor groups. These factors may include the presence of other growth factors and posttranscriptional modifications of CTGF.

### CTGF as a tumor promoter

Several potential functions for CTGF in tumor development in the group A tumors involve both direct action on the tumor cells and modification of tumor microenvironment. CTGF, in this manner, encourages tumor invasion, cell survival and angiogenesis.

Metastases formation requires that the cell detach from the matrix and adopt cytoskeltal changes reminiscent of mesenchymal cells. Normally, detachment from ECM results in cell death, which must also be avoided for tumor development. The process of epithelial to mesenchymal transition (EMT), seen during embryonic development, describes the process where epithelial cells acquire a mesenchymal cell phenotype, which is more prone to migrate and invade. EMT is also thought to be a key component in both malignant epithelial tumor progression [15] and fibrotic diseases [16]. Transforming growth factor-beta (TGF-β), and downstream mediators including CTGF are major initiators of EMT processes in both fibrosis [16] and cancer progression [17]. These processes may be independent from each other as a TGF-β and CTGF-dependent induction of EMT was independent of the profibrotic effects of TGF-β in proximal tubular cells [18]. CTGF has been implicated in other processes requiring cell migration such as the reepithelialisation in corneal epithelial cells, which is mediated through TGF-β and CTGF signaling, with downstream ERK1/2 and p38 MAPK activation [19].

A central part of CTGF's role in endothelial cell function and angiogenesis may involve direct interaction with integrins in mediating cell migration and to avoid cell death. Integrins anchor cells to the extracellular matrix and are involved in defining cell shape and motility and are even implicated in regulating cell cycle. CTGF stimulates integrin expression and is shown to bind directly to endothelial cell surface integrins [20,21] to mediate ECM interaction and prolonging cell survival. CTGF mediates anchorage-independent cancer cell growth and support for this is based on findings where anti-CTGF treatment inhibits anchorage-independent growth in vitro, primary tumor growth in vivo and macroscopic lymph node metastases [22].

CTGF is capable of up-regulating both matrix metalloproteinases (MMPs) and their inhibitors (tissue inhibitor of metalloproteinases, TIMPs) and has the potential to activate both the synthesis and degradation of the extracellular matrix. Some tumors involve a desmoplastic reaction, a process that closely resembles chronic fibroproliferative disease that may serve to protect tumor cells from immune detection and chemotherapy. CTGF involvement in this process has been demonstrated in pancreatic cancer [23].

Hypoxia is a strong inducer of CTGF expression. CTGF co-localizes with hypoxia marker CAIX and the CTGF promoter contains a recognition site for hypoxia growth factor, HIF [23,24]. In several systems, CTGF protects from hypoxia-induced apoptosis allowing tumor growth despite hypoxia [11,25]. The prolonged cellular survival may be mechanistically similar to CTGF treatment of mesangial cells, which leads to rapid activation of ERK1/2 and reduced phosphorylation of Bcl-2 promoted cell survival [26]. CTGF protects from hypoxia-induced apoptosis [23] and is a strong stimulator of angiogenesis [11]. In contrast, some studies have reported CTGF to induce tumor cell apoptosis but it has been argued that this may be due to either overexpression or very high doses of the protein [6].

### CTGF as a tumor suppressor

In some tumors (group B), CTGF may act as a tumor suppressor gene. In these cases, CTGF is often expressed in the normal cells and its expression decreases with the grade of malignancy. CTGF action in some tumors is also in sharp contrast to what is seen in others. For example, in lung cancer, CTGF appears to be acting through inhibition of phosphorylation of ERK1/2 [13,14,27]. CTGF induced suppression of ERK1/2 phosphorylation is also seen in ovarian cancer cells (Group C) [14]. In breast cancer cells (group A), however, CTGF activates ERK1/2 phosphorylation [28]. The β-catenin pathway also appears to react in opposite directions to CTGF stimulation in the different tumor groups and a paradoxal relationship between CTGF, β-catenin and cell migration is noted between tumors in the groups. For example, decreasing CTGF levels by silencing RNA in colorectal cancer cells (group B) increases their invasive ability and metastatic capacity in mice and over-expression of CTGF decreases β-catenin/T-cell factor signaling [29]. In esophageal carcinoma cells (group C), however, CTGF over-expression gave increased tumor formation in mice and this is shown to be via increased β-catenin/T-cell factor signaling [30].

### The role of CTGF in endocrine tumors

Endocrine tumors are rare hormone producing tumors derived from endocrine cells in many different organs in the body. Their common endocrine nature is the reason they are often grouped for study. They display, however, wide variations in symptoms, malignant potential and tumor biology and are classified according to the WHO classification scheme which sorts tumors according to primary tumor site, metastases development, hormone production and proliferation [31]. Our studies on CTGF expression in these tumors have identified clear differences in CTGF expression (See Additional file 1, group D).

#### *CTGF in ileal carcinoids*

Ileal carcinoids, also known as midgut carcinoids, are serotonin producing endocrine tumors found in the small intestine and proximal colon. These tumors are particularity interesting as they ubiquitously express very high levels of CTGF [32] and provide a platform for understanding CTGF function. Ileal carcinoids demonstrate several phenotypes potentially related to CTGF, namely: high level of cell migration, a reactive and well vascularised myofibroblast rich stroma and fibrosis development locally as well as in distal organs.

Ileal carcinoids have a pronounced association with fibrosis development [33-35]. Fibrosis caused by associated factors is emerging as a major issue in the morbidity and mortality of the disease [36,37]. Complications related to fibrosis arise in 16-48% of patients [35]. Tumor produced hormones, serotonin [38-41] and tachykinins [42-44], have been shown to be related to fibrosis. Growth factors such as PDGF, IGF-I, EGF, TGF-α and TGF-β and their receptors have also been discussed in the context [35,45-50]. TGF-β induced fibroblast proliferation, collagen synthesis and myofibroblast differentiation is mediated by CTGF dependent pathways [9,51,52]. Our work demonstrates that while myofibroblasts were found in various types of endocrine tumors, ileal carcinoids displayed exceptionally high proportion of α-SMA immunoreactive fibroblast-like cells in an extensive stroma.

The association between serotonin and CTGF is evident in the literature. Serotonin induction of CTGF expression in renal mesangial cells is accompanied by activation of serotonin receptor 5HT2A [53]. Serotonin is also implicated in CTGF induction in hepatic stellate cells and may have a function in the development of liver fibrosis [54]. A recent study in the KRJ-1 cell-line (derived from ileal carcinoid tumor) demonstrated that 5HT2B receptors are highly expressed in these cells compared to normal enterochromaffin cells. Blocking 5HT2B receptors with specific antagonists decreased ERK1/2 phosphorylation, reduced serotonin secretion, TGF-β, CTGF and FGF2 transcription and reduced proliferation [55]. Serotonin administration induces heart valve fibrosis that resembles carcinoid heart disease in rats through induction of TGF-β and CTGF in the heart smooth muscle [39]. Full length CTGF can be found in patient serum and is potentially involved in carcinoid associated fibrotic disease [56].

Ileal carcinoids maintain a high grade of differentiation and low proliferation. Despite this, they are truly malignant; most patients have metastatic disease at diagnosis and those who initially undergo "radical" surgery are frequently (50-80%) diagnosed with metastatic recurrence within 5-15 years [57-59]. There is no capsule surrounding the primary tumor, instead the tumor is comprised of small tumor clusters that often appear to spread laterally along the basal membrane and infiltrate the muscle layers. The histopathological appearance supports the hypothesis that cell migration is an early event in tumor development. CTGF is, as discussed earlier in the text, involved in both cell migration and EMT. It is feasible that the malignant behavior seen in ileal carcinoids may be in some ways related to the high degree of CTGF expression.

It is generally presumed that ileal carcinoids are derived from serotonin producing enterochromaffin (EC) cells in the small intestine. Using double staining techniques, we have recently shown that while EC cells in the stomach are CTGF negative, both CTGF-positive and negative fractions of EC cells are found in the intestine [60]. The function of CTGF in intestinal EC cells is unknown and may hold clues for understanding ileal carcinoid biology.

#### CTGF in enterochromaffin-like cell carcinoids

While the enterochromaffin-like cell carcinoids (ECL-CCs) displayed CTGF expression far lower than the ileal carcinoids, we were intrigued by the variation in expression between different types of ECL-CCs and the finding that CTGF immunoreactivity was strongest in tumor cells facing the fibrovascular stroma. CTGF expression is absent in normal enterochromaffin-like (ECL) cells and in foci of ECL-like cell hyperplasia. CTGF expression shows up first in the type I ECL-CCs larger than 5 mm, and its expression was correlated with a tumor size >1 cm. CTGF immunoreactivity in large type I ECL-CCs may be related to hypoxia, which occurs when tumor size exceeds the distance oxygen can diffuse into the tissue. CTGF is induced by hypoxia and is known to be a potent angiogenic factor (see above). CTGF and advanced tumor stage in well-differentiated ECL-CCs correlated but no correlation with serotonin expression was found. These results suggest that CTGF may be involved in initiating or promoting the neoplastic process. CTGF expression was absent in gastric poorly differentiated endocrine carcinomas (PDECs), and this may imply that CTGF is mainly involved in early pathogenesis becoming less important in the presence of further genetic aberrations and loss of differentiation.

CTGF is not correlated to proliferation in ileal carcinoids whereas, in ECL-CCs, the correlation between CTGF expression and Ki-67 proliferative index is negative [32,60]. We hypothesize that CTGF expression in well-differentiated tumors, in addition to proposed tumor promotion effects, has a simultaneous function in maintaining the low proliferative and differentiated phenotype. Loss of CTGF expression in more advanced, anaplastic stages supports this.

#### CTGF in endocrine tumors - concluding comments

Our observations can be summarized as follows; CTGF is highly expressed in a subgroup of well-differentiated endocrine tumors. In these tumors, proliferation is generally low and the endocrine phenotype is apparent. CTGF is often, but not always, in conjunction with serotonin production and in cases where high expression of both is present, extensive myofibroblast-rich stroma is found. Abundant expression of CTGF in some tumor types seems to correlate with increased size and tumor stage. We believe that CTGF may be involved in early neoplastic processes that allow for advanced tumor stage and metastases development. In contrast to this, CTGF expression in the highly proliferative, PDEC is most often lost [32,60]. CTGF expression in normal endocrine cells has not been systematically studied in all organs. Our results in endocrine tumors indicate a complex relationship between CTGF and tumor progression but should be verified in other tumor cohorts. For these reasons, we have chosen not to place these tumors in the categories A-C but in a separate group, D, until there is enough data for these tumors to be sorted with certainty.

### CTGF as a treatment target

Regulating the TGF-β pathway has long been a goal for treatment of both cancer and fibrotic disease. TGF-β signaling is however more complicated than initially thought and strategies that block downstream events in the cascade may offer more selective effects. CTGF is potentially an important therapy target. Our understanding of its functions is still in the early stages and more knowledge is needed about its processing in vivo to reliably predict its function in vivo. CTGF has been shown to bind several receptors but there is to date no known CTGF-specific receptor.

Results from animal models of cancer where CTGF signaling is blocked have shown promise. CTGF-specific antibodies (FG-3019) inhibit pancreatic tumor growth and metastasis in nude mice [22] as well as in an orthotopic mouse model [61]. The main anti-tumoral effects were increased tumor cell apoptosis and attenuated angiogenesis. Clinical trials using FG-3019 in patients with pancreas cancer are now initiated. Another approach involves a DNA vaccine consisting of a combination of CTGF and viral tumor antigen E7 DNAs. This vaccine was shown to generate T-cell mediated antitumor immune response in a mouse cervical cancer [62]. Synergistic benefits of combining the CTGF DNA vaccine in combination with chemotherapy is reported where antitumor effects included inhibition of angiogenesis [63].

The potential tumor suppressive effect of CTGF should not, however, be forgotten. Based on our current understanding, there is a risk that treatment which results in down regulation of CTGF may also remove its simultaneous tumor suppressive effects. Under this concept, there is an additional risk of stimulating development or progress of an additional tumor in another organ where CTGF has a tumor suppressive effect. This risk may be a limiting factor when deciding to treat other tumor forms with better prognosis than pancreas cancer.

## Conclusions

CTGF in the context of cancer is a clearly intriguing molecule and a potential candidate for therapeutic targeting. A full characterization of its regulation, in particular, understanding the determinates of its opposite actions in different cell-lines and cancer types is essential for predicting and preventing paradoxal tumor reactions.

## Competing interests

The authors declare that they have no competing interests.

## Authors' contributions

AJ and JLC have 1) made substantial contributions to conception and design, or acquisition of data, or analysis and interpretation of data; 2) have been involved in drafting the manuscript or revising it critically for important intellectual content; and 3) have given final approval of the version to be published.

## Supplementary Material

Additional file 1 **Summary of current knowledge concerning CTGF expression in tumors and corresponding normal tissues in relation to tumor progression**. Click here for file
