# Supplementary material for: Connective tissue growth factor in tumor pathogenesis
Source: Fibrogenesis Tissue Repair. 2012 Jun 6;5(Suppl 1):S8. doi: 10.1186/1755-1536-5-S1-S8 (PMC3368788; doi:10.1186/1755-1536-5-S1-S8)
Supplement: Additional file 1 — Summary of current knowledge concerning CTGF expression in tumors and corresponding normal tissues in relation to tumor progression. [file 1755-1536-5-S1-S8-S1.rtf]

Table 1: Summary of current knowledge concerning CTGF expression in tumors and corresponding normal tissues in relation to tumor progression


Group A — Cancers with positive correlation between CTGF expression and tumor progression

Cancer type	
Normal CTGF†	
Proposed mechanism of CTGF. Correlation to tumor progression.	

Acute lymphoblastic leukemia (ALL)	
Absent	
High CTGF mRNA expression (in leukemic cells of B-lineage compared to normal hematopoetic and non-ALL) correlated to worsening of survival and to unfavorable cytogenetics (69). Three other studies confirm an exceptionally high CTGF mRNA in the pre-B-cell lineage in ALL, ranging from 61-75% of the cases (70-72). Those who lacked CTGF expression belonged to subgroup with E2A-PBX1 translocation (72).	

Breast cancer	
Absent	
A positive association is found between high CTGF mRNA expression, tumor size and stage when comparing primary breast tumors with normal breast tissues (73). CTGF expression is high in human breast cancer cell line subpopulations with elevated osteolytic bone metastatic activity (74). High CTGF IR** is inversely associated with a positive chemotherapy response in human breast cancers. In cell cultures, over-expression of CTGF mediates this drug-resistance effect via up-regulation of Bcl-xL and cIAP1 and activation of ERK1/2 and apoptosis resistance (28). Treatment with a CCN2/CTGF-neutralizing antibody greatly decreased osteolytic bone metastasis and microvasculature in a murine model of breast cancer (75). 	

Cervical cancer 	
Absent	
CTGF mRNA is differentially uppregulated in late stages of cervical cancer when compared to early stages (76).	

Gastric carcinoma 	
Low or Absent	
High CTGF IR is correlated to shorter survival in gastric cancer. CTGF is found to be an independent predictor of poor prognosis and increased lymph node metastases (77). The expression of CTGF correlated positively to expression of VEGF, VEGF-C and VEGF-D (78).	

Glioma	
Low or Absent	
High CTGF mRNA expression is detected in 58% of primary gliomas, and correlates to poor patient survival (79).
	

Hepato-cellular carcinoma (HCC) 
	
Low or Absent	
CTGF mRNA is elevated in HCC compared to pre-cancerous and normal liver tissues, and correlates to recurrence and metastasis. The more aggressive nodular subtype of HCC has higher CTGF mRNA levels, less differentiation and increased liver cirrhosis in comparison to solitary large HCC (80). Serum CTGF is elevated 6-fold in HCC patients compared to healthy controls and correlates positively to higher tumor stage, more venous invasion and shorter survival. Moreover, a positive correlation is found between serum concentrations of CTGF and VEGF and bFGF, indicating a role in tumor angiogenesis (18). 	

Pancreatic adeno-carcinoma	
Low or Absent
	
CTGF IR is stronger in 70% of pancreatic cancer, compared to normal pancreas from same individuals, and it co-localised with hypoxia. Mechanistic studies in cell lines and a mouse model indicate that tumor-cell derived CTGF protected from hypoxia-induced apoptosis (23).	


Group B — Cancers with negative correlation between CTGF expression and tumor progression

Cancer type	
Normal CTGF	
Proposed mechanism of CTGF. Correlation to tumor progression.	
Chondro
sarcoma	
Yes	
Low CTGF expression correlates to poor survival and higher tumor stage (12).	

Colorectal cancer (CRC)	
Yes
	
Patients with low CTGF expression had shorter survival time.  In vitro studies show that decreasing CTGF levels by silencing RNA in CRC cell lines increases their invasive ability and metastatic capacity in mice. Overexpressoin of CTGF decrease β-catenin/T-cell factor signalling and its downstream effector gene MMP 7 (29) .	

Lung cancer
(non small cell)	
Yes
	
CTGF mRNA and IR are down-regulated in 65% of primary lung cancers compared to the paired normal lung tissues. Low CTGF levels correlate with high tumor stage and metastasis (13). In vitro studies show that CTGF suppressed lung cancer cell growth by induction of p53 and overexpression of CTGF inhibit of IGF-I dependent Akt phosphorylation and EGF-dependent ERK1/2 phosphorylation (27) (25).	

Meningioma  	
Yes	
Meningiomas are most often benign but loss of chromosome 6q is associated with malignant progression. CTGF (6q23) mRNA levels are lower in recurrences compared to primary tumors (81). 	


Group C — Cancers with complex correlation between CTGF expression and tumor progression

Cancer type	
Normal CTGF	
Proposed mechanism of CTGF. Correlation to tumor progression.	
Esophageal carcinomas	Yes
 	Increased TGF-β and CTGF expression in majority of esophageal cancer in comparison with normal controls. The degree of fibrosis is different in squamous (ESCC) and adenocarcinomas and is significantly related to CTGF mRNA levels. The presence of CTGF in ECSS is associated with longer survival, whereas in adenocarcinomas it influenced survival negatively (82). In contrast, high CTGF IR in ESCC was related to poor survival and in vitro studies show potential involvement of CTGF in Fascin-mediated proliferation and invasiveness, via TGF-β-pathway (83). Moreover, high CTGF mRNA levels in pairwise ESCC samples correlated to more metastasis and higher tumor grade. CTGF over-expression in ECSS cells increased tumor formation in mice and activation of β-catenin and TCF/Lef signaling (30).	
Gallbladder cancer (GBC)	Low 	CTGF mRNA and IR is overexpressed in primary GBC, but not in metastatic GBC, compared with non-neoplastic gallbladder epithelium. GBCs (36%) with high CTGF IR have a favorable survival (84).	
Ovarian cancer	Yes
	In ovarian cancers, CTGF IR varies. In the earlier stages before metastases development (stage I and II) of ovarian cancer CTGF methylation is frequent and low CTGF IR correlates to lower survival rates. CTGF may initially function as a tumor suppressor gene. In advanced stages (III and IV), CTGF expression is often restored and may contribute to the more malignant phenotype. Homozygous loss of CTGF is seen in ovarian cancer cell lines. In an ovarian cancer cell line, CTGF treatment suppresses EGF-induced phosphorylation of ERK1/2.  (14).	
Wilms tumor	Yes	CTGF is over-expressed in a subset of tumors, compared to normal kidney tissue. CTGF is activated in nephrogenesis and early tumorigenesis, while its expression decreases with tumor progression (85).	
Group D — Endocrine tumors with complex or unknown correlation between CTGF expression and tumor progression

Cancer type	
Normal CTGF	
Proposed mechanism of CTGF. Correlation to tumor progression. 	

ECL-CCs§	
Absent	
CTGF-IR is often absent in small primary ECL-CCs without metastatic spread (stage 1). Expression correlates with tumor size >1 cm in type I ECL-CCs and to advanced stage in the well differentiated ECL-CCs. CTGF is mainly absent in poorly differentiated ECL-CCs (32, 65).	

Ileal carcinoid	
Var*	
Very high CTGF IR is present in all well differentiated malignant tumors. CTGF-IR is lower or absent in poorly differentiated ileal carcinoids (32, 65).	

Appendix carcinoid	
Var*	
Low CTGF-IR in tumors (32).	

Hindgut 	
Var*	
Often express high levels of CTGF-IR with unknown significance (32).	

Endocrine pancreatic tumor	
nd#	
Variable CTGF-IR with unknown significance. Insulin producing tumors do not express CTGF (32).	

Lung carcinoid	
nd#	
Variable CTGF-IR with unknown significance (32).	

Follicular thyroid adenoma and cancer	
nd#	
Some follicular thyroid adenomas express very low levels of CTGF-IR. Follicular thyroid cancer displayed negative CTGF-IR (32).	

Medullary thyroid cancer	
nd#	
Low CTGF-IR is generally found (32).	

Pheocromocytoma	
nd#	
No CTGF-IR (32).	

Adrenocortical adenoma and cancer	
nd#	
No CTGF-IR (32).	

Papillary thyroid adenoma and cancer	
nd#	
No CTGF-IR (32).	
* Both CTGF-positive and negative fractions of endocrine cells are found in the gastric and intestinal mucosa, namely: enterochromaffin cells (EC) (serotonin-IR), L  (PYY- and GLP-1-IR), D (somatostatin-IR) and P/D1 (ghrelin-IR) cells (65). 
§ ECL-CCs: enterochromaffin-like cell carcinoids 
† CTGF expression corresponding normal cells or tissue
#nd: Not done;
 **IR: Immunoreactivity
